# Exploring Hydroxytyrosol as a Promising Virucidal Agent: In Silico and In Vitro Insights into Enveloped Viruses

**DOI:** 10.3390/cimb48050481

**Published:** 2026-05-05

**Authors:** Hanan El Ouadi, Zineb Rhazzar, Barbara Poddesu, Boutaina Addoum, Laila Benbacer, Franco Lori, Siham Fellahi, Davide De Forni, Omar Nyabi, Jean-Luc Gala, Elmostafa El Fahime, Saber Boutayeb, Lahcen Belyamani, Khalid Ennibi, Ouafaa Fassi Fehri, Nadia Touil

**Affiliations:** 1 Mohammed VI Center of Research and Innovation (CM6RI), Rabat 10100, Morocco; melfahime@cm6.ma (E.E.F.); sboutayeb@cm6.ma (S.B.); belyamani@gmail.com (L.B.); 2 Microbiology, Immunology, and Infectious Diseases Unit, Department of Pathology and Veterinary Public Health, Institut Agronomique et Vétérinaire Hassan II, Rabat 10100, Morocco; o.fassifihri@iav.ac.ma; 3 Molecular Virology and Onco-Biology, Faculty of Medicine and Pharmacy of Rabat, Mohammed V University of Rabat, Rabat 10100, Morocco; zineb.rhazzar@um5r.ac.ma (Z.R.); kennibi@yahoo.fr (K.E.); 4 Biomedical and Epidemiology Research Unit (URBE), Center for Virology, Infectious and Tropical Diseases, Mohammed V Military Training Hospital, Rabat 10100, Morocco; 5 ViroStatics srl, Viale Umberto I, 46, 07100 Sassari, Italy; b.poddesu@virostatics.com (B.P.); f.lori@virostatics.com (F.L.); d.deforni@virostatics.com (D.D.F.); 6 Biology and Medical Research Unit, National Center for Energy, Nuclear Sciences and Techniques, Rabat 10010, Morocco; addoumboutaina72@gmail.com (B.A.); lbenbacer@yahoo.com (L.B.); 7 Department of Veterinary Pathology and Public Health, Hassan Institut Agronomique et Vétérinaire Hassan II, B.P. 6202, Rabat 10000, Morocco; s.fellahi@iav.ac.ma; 8 Center for Applied Molecular Technologies (CTMA), Institute of Clinical and Experimental Research, Université Catholique de Louvain, 1200 Brussels, Belgium; omar.nyabi@uclouvain.be (O.N.); jean-luc.gala@uclouvain.be (J.-L.G.); 9 Departement LARMIAS, Mohammed VI University of Sciences and Health (UM6SS), Casablanca 20370, Morocco

**Keywords:** hydroxytyrosol (HT), virucidal activity, enveloped viruses, non-enveloped viruses, antiviral in silico and in vitro approaches

## Abstract

The research investigates synthetic hydroxytyrosol (HT) antiviral properties against enveloped and non-enveloped viruses using in silico and in vitro methods. Molecular docking and ADMET analyses suggested favorable interactions of HT with ceramide and sphingomyelin (binding energies of −6.0 and −5.9 kcal/mol, respectively). Favorable predicted pharmacokinetics and safety profiles were also observed. In vitro tests provided preliminary evidence of the dose- and time-dependent virucidal effect of HT against several enveloped viruses, including HSV-1, West Nile virus, SARS-CoV-2 and various influenza A subtypes, which resulted in substantial viral load decreases at 1000 µg/mL. The viral titer of the measles virus decreased by 4.62 log_10_ units during the 2 h of exposure. No virucidal activity was observed against the non-enveloped bovine rotavirus. Overall, these findings suggest that hydroxytyrosol may represent a promising candidate for further investigation as a virucidal agent, particularly against enveloped viruses.

## 1. Introduction

Hydroxytyrosol (HT), a potent polyphenol naturally found in olive oil and olive leaves (*Olea europaea*), has various bioactivities, including antioxidant [1], anti-inflammatory [2], anti-cancer [3] and antimicrobial [4] activities against bacteria, fungi, and mycoplasmas. The antiviral activity of HT has been reported against many viruses, with a specificity for enveloped ones, such influenza and HIV viruses [5]. Researchers have proven that HT effectively lowered titers of enveloped viruses in a dose-dependent manner, while non-enveloped viruses conserved their titers when tested with HT. The specificity supposes that the mechanism of the antiviral effect of HT requires the presence of the viral envelope [5]. These observations were initially based on natural HT extract; in the present work, we aimed to confirm this resistance pattern using synthetic, highly purified HT and to determine whether this lack of activity is specific to non-enveloped viruses. To answer this question, we studied bovine rotavirus. Nowadays, HT is being investigated for its ability to inhibit viruses’ progression. This compound may exert its effect by interacting with lipid rafts and membrane microdomains enriched with sterols and sphingolipids, which are crucial for viral entry and fusion processes [6]. In contrast to previous studies based on natural hydroxytyrosol extracts, which may contain multiple phenolic compounds and confounding bioactive substances, the present study employed highly purified synthetic hydroxytyrosol (≥98%). This approach allowed us to specifically assess the intrinsic virucidal activity of hydroxytyrosol independently of plant-derived impurities, thereby improving the reproducibility and enabling a more accurate interpretation of its antiviral properties.

Entry processes of enveloped viruses associated with membrane rafts have been evaluated for many viruses such as coronaviruses (including SARS-CoV-2), Ebola virus, Marburg virus, West Nile virus (WNV), Japanese encephalitis virus (JEV), herpes simplex virus 1 (HSV-1), human immunodeficiency virus (HIV), Semliki Forest virus, and others [7]. Most enveloped viruses release their internal genomes and proteins into the cell’s interior via fusion events triggered by viral surface proteins interacting with cellular and viral membranes, occurring right after the virus attaches to receptors or enters through the endocytic route [7,8].

Membrane rafts are small (10–200 nm), heterogeneous, highly dynamic, sterol- and sphingolipid-enriched domains that are pivotal in organizing and compartmentalizing various cellular processes [9,10]. These domains concentrate on viral components and receptors, facilitating viral entry and replication [8]. In terms of viral replication, membrane rafts are not strictly necessary for virus replication, but they likely enhance the efficiency of viral entry, genome replication, and the production of infectious virions. Furthermore, these lipid microdomains are integral to the activation of cellular signaling pathways that benefit viral replication, thereby promoting the virus’s ability to infect and proliferate [7,8]. Since non-enveloped viruses lack membrane lipids and sphingolipid-rich microdomains, modeling of lipid raft interaction does not apply to rotaviruses, further highlighting the structural basis underlying their resistance to HT. Despite this, an in vitro study appears necessary to confirm the hypothesis.

Recent epidemiological in vitro and computational reports highlight the protective effect of HT. It reduces the serum lipids in mice fed with high-cholesterol diets, indirectly modifying the composition of their plasma membrane [11]. Furthermore, HT improves endothelial dysfunction, decreases oxidative stress, and is neuro- and cardio-protective. Due to all these biological properties, HT is currently one of the most actively investigated natural phenols, with great pharmacological potential [12]. This membrane dependence provides a mechanistic hypothesis for the selective activity of HT against enveloped viruses.

HT demonstrates broad-spectrum antiviral activity, particularly against enveloped viruses like influenza A (including subtypes H1N1, H3N2, H5N1, and H9N2), HIV, and coronaviruses [5]. It may, for instance, induce morphological changes that reduce the infectivity of the influenza virus. While the exact mechanism remains unclear, it seems to be dependent on the presence of the viral envelope [5]. Given the complexity of these interactions and the lack of detailed mechanistic understanding, many authors have directed their research towards in silico studies to better explore and model the potential antiviral effects of HT. These computational approaches provide a valuable tool for investigating the molecular dynamics of HT–virus interactions, offering insights that are difficult to obtain through traditional experimental methods alone. However, these studies were based on natural HT preparations, leaving open the question of whether purified synthetic HT would exhibit the same selectivity.

To our knowledge, no studies have specifically reported the virucidal activity of highly purified synthetic hydroxytyrosol against West Nile virus (WNV), measles virus (MeV), and HSV-1. The present study provides preliminary evidence supporting the virucidal activity of synthetic hydroxytyrosol against these viruses and suggests an extension of the antiviral spectrum previously associated with hydroxytyrosol. In addition, our findings are consistent with previous reports describing virucidal activity against enveloped viruses such as human influenza A (H1N1), avian influenza (H5N1), and SARS-CoV-2. Notably, the lack of virucidal activity observed against bovine rotavirus (BRV) further supports the hypothesis that the activity of hydroxytyrosol is influenced by viral envelope-associated structural features.

## 2. Materials and Methods

### 2.1. Molecular Modeling Studies

The following section describes the molecular modeling procedure, but it is essential to understand that this study used in silico methods only for enveloped viruses. Molecular modeling and lipid interaction modeling require membrane lipids composed of sphingomyelin, ceramide, cholesterol, and phosphatidylinositol. The structure of rotaviruses does not include lipid bilayers or sphingolipids [13], which prevents their use in lipid-based molecular modeling. Computational evaluations were performed with hydroxytyrosol molecules to study their native molecular interactions without interference from the contaminants present in natural extracts.

#### 2.1.1. Software

A comprehensive in silico analysis was employed for this study by utilizing a variety of bioinformatics tools, including software databases and online libraries, as reported in Table S1 (Table S1 is provided in the Supplementary Materials).

#### 2.1.2. Ligand System

Hydroxytyrosol is a small natural phenolic compound found in olives and their derivatives (molecular weight of 154.16 g/mol). The 2D/3D structure of this molecule was retrieved from the PubChem database (https://pubchem.ncbi.nlm.nih.gov) in SDF format and converted to PDB format using the Open Babel Open Babel 2.4.1, GUI program. The ligands were then prepared for docking by optimizing their geometries and assigning Gasteiger charges. The characteristics of the HT are summarized in Table S2 according to the Pubchem and Drugbank data (Table S2 is provided in the Supplementary Materials).

#### 2.1.3. Lipid Rafts as Target for Viral Inhibition

Lipid rafts are distinct lipid domains in the external leaflet of the plasma membrane and are rich in glycosphingolipids, cholesterol, glycosylphosphati-dylinositol-anchored proteins and signaling proteins [10]. They take part actively in a variety of molecular processes, like disruption of viral infections, signal transduction, cell-to-cell communication, immune response initiation, membrane transport and apoptosis [7]. Our methodological approach will underline the main role of lipid rafts at viral life cycles.

#### 2.1.4. Protein System

The target protein structures were selected based on literature data and retrieved from the Protein Data Bank (www.rcsb.org), as reported in Table S3 (Table S3 is provided in the Supplementary Materials). To optimize the protein structures for docking, all polar hydrogen atoms were added using the BIOVIA Discovery Studio Visualizer (version 2020) to reduce structural tension and enhance the docking accuracy. The protein structures were energy-minimized in a vacuum environment, allowing hydrogen atoms to move while keeping heavy atoms fixed in their crystal positions. Protein and ligand preparations were performed using AutoDockTools (ADT). Gasteiger charges were computed, and nonpolar hydrogen atoms were merged with their respective carbon atoms. The final PDBQT files were generated for a molecular docking analysis.

#### 2.1.5. Molecular Docking

This section aims to assess the antiviral potential of HT through in silico molecular docking analyses. To this end, the binding affinity of HT was evaluated against the catalytic sites of seven key molecular targets involved in viral entry and membrane fusion. To perform our molecular docking simulations, we used AutoDockVina 1.1.2 within the PyRx virtual screening software. The protein structure was prepared in the PyRx 0.8 version software, resulting in a PDBQT file containing hydrogen atoms for all polar residues. Rotatable bonds were assigned to the ligands and simulated the interaction L-P; then, scoring calculations were performed using the Lamarckian genetic algorithm (LGA) method. The docking site on the selected protein target was defined by establishing a grid box with dimensions of (X, Y, Z): 81 Å × 61 Å × 64 Å, and a grid spacing of 0.375 Å was positioned at the binding pocket of the seven targets. The best conformation, determined by the lowest docked energy, was selected after completing the docking search. Ten runs with AutoDockVina were executed for each ligand structure, with the best pose saved for each run. The final affinity value was determined by averaging the affinities of the best poses. Subsequently, each molecule and its associated RMSD were arranged by the PyRx software in ascending order based on their scores to select the lead derivatives. The 2D interactions between the HT complex and the protein conformations, including hydrogen bonds and bond lengths, were analyzed using the Biova studio visualizer 2024 (v24.1)

#### 2.1.6. ADME/ToxPredictions

During this in silico process, it was imperative to identify the ADMET properties of HT by evaluating toxicity through the measure of absorption, distribution, metabolism, excretion, and toxicity. Also, key toxicity endpoints such as the hepatotoxicity and oral rat acute toxicity (LD_50_) were selected for assessment. The LD_50_ is the cornerstone of this pharmacokinetic screening, due to its capacity to determine the lethal dose of a compound, thereby facilitating classification based on the Globally Harmonized System (GHS) and predicting the extent of toxicity.

To assess the pharmacokinetic properties of the studied ligands, in silico ADME (absorption, distribution, metabolism, and excretion) predictions were performed using the SwissADME web server (http://www.swissadme.ch/ (accessed on 15 January 2026)). Additionally, toxicity and metabolism analyses were conducted using ADMETlab 2.0 (https://admetmesh.scbdd.com/service/evaluation/cal (accessed on 15 January 2026)), providing insights into the drug likeness and safety profiles of the ligands.

### 2.2. In Vitro Studies

#### 2.2.1. Compounds

Synthetic hydroxytyrosol (HT, purity of ≥98%) was provided by Nova Mentis. The compound was stored at +4 °C. A stock solution was prepared fresh for each experiment in water at the appropriate concentration.

An antibiotic (penicillin/streptomycin), L-glutamine, trypsin–EDTA (0.25%), bovine serum albumin (BSA at 7.5%), bovine donor serum (BDS), fetal calf serum (FBS), penicillin (100 IU/mL), streptomycin (100 µg/mL) and Dulbecco’s modified Eagle’s medium (DMEM) were supplied by Gibco BRL (Grand Island, NY, USA) and Biowest (Nuaillé, France). Stock solutions of the TPCK (Sigma, St. Louis, MO, USA) (2 mg/mL) were made in sterilized water.

#### 2.2.2. Cells and Viruses

Vero E6 cells, Vero cells (Cercopithecus aethiops kidney, ATCC CRL-1586 and CCL-81), MA104 cells (ATTC, CRL-2378.1™) and MDCK cells (Madin–Darby canine kidney, ATCC CCL-34) were cultured in Dulbecco’s modified Eagle’s medium (DMEM) supplemented with 10% FBS, a 1% antibiotic solution and 1% L-glutamine, i.e., a complete medium, at 37 °C with 5% CO2.

The human 2019-nCoV strain 2019-nCoV/Italy-INMI1, originally isolated in Italy from a clinical specimen collected on 29 January 2020 (following its emergence in China), was kindly provided by the Instituto Lazzaro Spallanzani, Rome, Italy (GenBank accession number MT066156.1, and GISAID accession number EPI_ISL_410545). The live highly pathogenic avian influenza virus H5N1 (A/ck/Israel/65/10) was obtained by the Animal and Plant Health Agency, Surrey, UK, and is available via the European Virus Archive (accessed on 12 June 2024 at https://www.european-virus-archive.com/virus/live-highly-pathogenic-avian-influenza-h5n1-virus-ackisrael6510). The human influenza A H1N1 virus (Wisconsin/67/22) was purchased from Zeptometrix (Cat.N°. 0810685CF). The avian influenza A virus [A/chicken/Morocco/469/2023(H9N2)], corresponding to a partial coding sequence deposited under GenBank accession number OR149149.1, was obtained from the Avian Pathology Department of the Agronomy and Veterinary Institute Hassan II, Rabat Morocco. The virus was subsequently adapted to MDCK cells at passage 3 (P3). The measles virus (MeV) strain MVi/Rabat.MAR/29.24/[B3], belonging to genotype B3 (GenBank) strained ion number PQ069064.2, was isolated using Vero/DogSLAM and further adapted to passage 6 (P6) on Vero cells. The West Nile virus strain (WNV) was kindly provided by Pr. Fassi Fihri from the Epidemiology/Virology Infectious Department of the Agronomy and Veterinary Institute Hassan II, Rabat Morocco. Herpes simplex virus type 1 (HSV-1) was adapted in Vero cells up to passage P8 following established protocols.

SARS-CoV-2 was propagated in Vero E6 cells, while the H5N1, H9N2 and H1N1 viruses were propagated in MDCK cells; the WNV, MeV and HSV-1 strains were spread in the Vero cell line and BRV was propagated in MA104 cells. Viral cultures of SARS-CoV-2, H1N1 and H5N1 were conducted in the Biosafety Level 3 (BSL-3) facility at the ViroStatics facilities located at the Scientific and Technological Park Porto Conte RicercheSrl (Alghero, Italy). The WN, MeV and HSV-1 viruses were manipulated in the BSL-3 level and BSL-2 facility of the Virology Center, Infectious and Tropical Diseases of Rabat, Morocco, Military Hospital of Rabat.

The BRV viral suspension, previously activated by incubation with sigma trypsin (10 µg/mL, 30 min at 37 °C), was added at a predefined multiplicity of infection (MOI) to the cell monolayer at 80% confluence, previously washed with PBS. After 1 h of adsorption at 37 °C with gentle agitation, the inoculum was removed and replaced with a maintenance medium (DMEM, 0% SFB, sigma trypsin 0.5–1 µg/mL). The cultures were then incubated at 37 °C and 5% CO_2_, and monitored daily for the appearance of a cytopathic effect. The rotavirus strain was supplied and handled in the BSL-3 level and BSL-2 facility of the Virology Center, Infectious and Tropical Diseases of Rabat, Morocco, Military Hospital of Rabat.

All viruses were propagated in cells in 2% FBS until 80% cellular lysis was observed. The supernatants containing the released viral particles were collected and centrifuged at 600× *g* for 5 min. The clarified supernatant was frozen and kept at −80 °C until use. The viral titer of each virus was determined by cytopathic effect determination through a Reed and Muench assay [14], and was given by the tissue culture infectious dose per ml (TCID_50_/mL), which is defined as the dilution of a virus required to infect 50% of a given cell culture.

#### 2.2.3. Cell Viability Assay

The effect of different concentrations of HT on the viability of cells was determined by a 3-[4,5-dimethyl thiazol-2-yl]-2,5-diphenyl tetrazolium bromide (MTT) assay. MDCK cells were grown (2.5 × 10^4^ cells/well) in 96-well plates for 24 h. The medium was replaced with DMEM containing different concentrations of component HT (0.1, 10, 100, 1000 and 10,000 µg/mL). Then, the cells were further incubated for 48 h. A negative control consisting of untreated cells cultured in DMEM alone was included under the same experimental conditions. After incubating the cells for a specific time at 37 °C, MTT (5 mg/mL in 1× PBS) was added to each well and the cells were incubated for 4 h. After removal of the supernatant, 50 µL of DMSO was added and incubated for 30 min. Next, the absorbance was recorded on a microplate reader at a wavelength of 540–600 nm [15].

#### 2.2.4. Virucidal Screening

**Figure 1:**
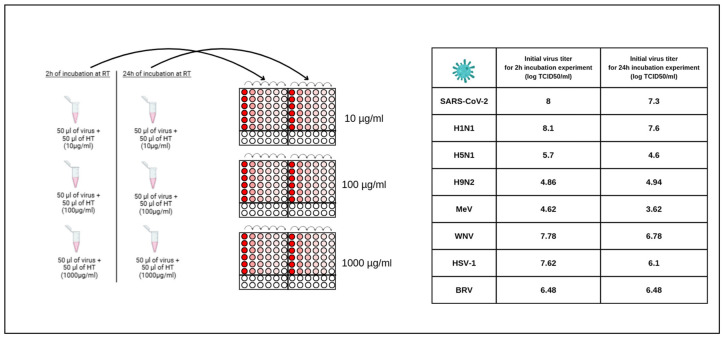
Detailed schema of workflow of in vitro screening of different HT concentrations on SARS-CoV-2, H1N1, H5N1, H9N2, MeV, WNV, BRV and HSV-1 viruses.

The virucidal activity of each virus consisted of incubating the virus with HT at room temperature. The BRV, SARS-CoV-2, H1N1, H5N1, H9N2, MeV, WN and HSV-1 viruses, with the initial titers shown in Figure 1, were mixed with HT concentrations (10–1000 µg/mL) in sterile tubes, for 2 h and 24 h. Then, the virus–HT mixture was collected to propagate in the respective host cells.

The cells were cultured in 96-well plates (2 × 10^4^ cells/well) with serial dilutions of each collected treated virus (10^−1^–10^−6^), in 96-well replicates run in parallel. The cytopathic effect (CPE) was observed daily to determine the viral titers through a Reed and Muench assay [14]. For BRV, a trypsin activation step (30 min at 37 °C) was performed after the virus–HT incubation and immediately prior to titration and inoculation onto MA104 cells.

## 3. Results

### 3.1. Molecular Modeling Studies

#### 3.1.1. Molecular Docking

**Figure 2:**
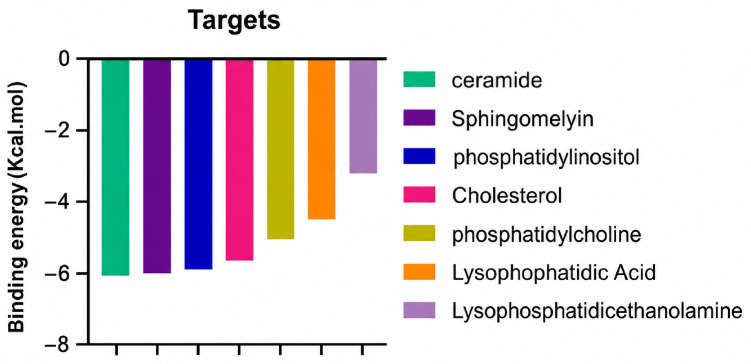
Comparative analysis of binding energy of all compounds obtained from PyRx.

A molecular docking analysis was conducted to evaluate the antiviral potential of HT. The binding affinity of HT was assessed within the catalytic site of seven key molecular targets associated with viral entry and membrane fusion. The docking analysis revealed significant interactions between HT and these targets, suggesting its potential inhibitory effects (Figure 2).

Interestingly, the docking analysis revealed that HT exhibited the strongest binding affinities with ceramide and sphingomyelin, two key components of lipid rafts, with respective binding energies of −6.0 and −5.9 kcal/mol.

#### 3.1.2. The 2D Structure of the Complex L-P

The results of the Pyrx docking studies indicated that the phytocompound HT can exert several potential hydrogen bonds (HBs) and non-bonding interactions with the core functional residues of the target proteins.

A binding energy analysis of all seven targets obtained is shown in Table 1. The obtained data indicate that, compared to other compounds, ceramide and sphingomyelin show the most favorable binding affinity with the HT ligand and can form strong hydrogen bond interactions with core functional amino acids, such as Tyr182 and Ile219 for ceramide, with a docking score of −6 kcal/mol, and Phe163 and Ser167 for sphingomyelin, with a binding energy of −5.9 kcal/mol.

The compound also exhibited Pi–Pi interactions with the Leu218 and Val234 amino acids near the catalytic site.

#### 3.1.3. ADME/Tox Calculation

HT exhibits favorable physicochemical and pharmacokinetic properties, supporting its potential as a drug-like molecule. With a molecular weight of 154.16 g/mol, two rotatable bonds, three hydrogen bond acceptors, and three hydrogen bond donors, it maintains a good molecular flexibility and hydrogen bonding capacity. Its topological polar surface area (TPSA) of 60.

**Figure 3:**
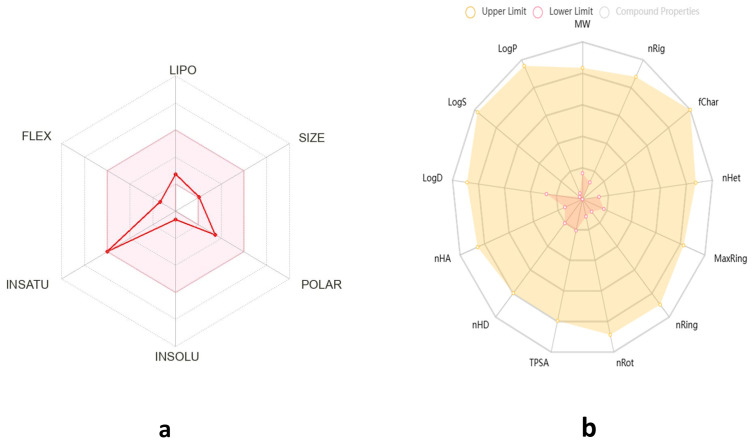
Pharmacokinetic behavior and toxicity prediction pie chart generated by SwissAdmet (**a**) and PreADMET (**b**).

69 Å^2^ suggests good membrane permeability. The compound has a moderate lipophilicity (Log Po/w = 1.28) and is classified as highly soluble in water (37.5 mg/mL; 0.243 mol/L), which enhances its bioavailability (Figure 3).

Pharmaco-kinetically, HT demonstrates high gastrointestinal absorption, but does not permeate the blood–brain barrier (BBB), limiting its potential for central nervous system applications. It is not a substrate for P-glycoprotein (P-gp) and does not inhibit key cytochrome P450 enzymes (CYP1A2, CYP2C19, CYP2C9, CYP2D6, CYP3A4), suggesting a low risk of metabolic drug interactions.

According to drug-likeness criteria, HT complies with Lipinski’s, Veber’s, and Egan’s rules, indicating a good oral bioavailability, though it violates the Ghose and Muegge filters due to its low molecular weight (Table 2).

Overall, HT presents promising pharmacokinetic attributes with a high solubility and absorption, making it a viable candidate for further drug development, particularly in non-CNS therapeutic applications.

As outlined in the methodology section, every molecule can undergo an assessment by uploading its bioavailability radar. In Figure 1, we depict the bioavailability radar for HT by using the bioinformatics tools identified in the methodology section.

The compound HT exhibits a limited permeability across biological barriers, as indicated by its poor Caco-2 permeability (−4.997) and negligible MDCK permeability (0), suggesting restricted passive diffusion and potential absorption challenges. Although human intestinal absorption (HIA) data are unavailable, its plasma protein binding (PPB) of 26.6% indicates a moderate level of systemic distribution. Blood–brain barrier penetration data are absent, reinforcing previous findings that HT is unlikely to access the central nervous system.

Metabolically, HT shows positive human liver microsome (HLM) stability, suggesting a reasonable half-life in hepatic metabolism. Toxicological assessments indicate a relatively low risk of nephrotoxicity (0.057), neurotoxicity (0.041), hematotoxicity (0.097), and genotoxicity (0.076), while moderate concerns arise regarding ototoxicity (0.553) and hepatotoxicity (0.377). Additionally, HT demonstrates a high likelihood of causing skin sensitization (0.992), eye corrosion (0.958), and eye irritation (0.998), necessitating caution in formulation and handling. The compound exhibits minimal interactions with nuclear receptors (NR-AhR, NR-AR, NR-ER, and NR-PPAR-gamma), but shows activation of the oxidative stress response pathway (SR-ARE), suggesting a potential role in oxidative stress modulation. Despite its favorable metabolic stability and low systemic toxicity, the compound’s poor permeability and potential for local irritation may limit its clinical applicability, warranting further structural modifications to enhance its drug-like properties.

Based on the Protox server, HT has a predicted LD_50_ of 2820 mg/kg, classifying it as toxicity class 5, which indicates a relatively low acute toxicity (Table 3).

### 3.2. In Vitro Studies

#### 3.2.1. Cell Viability

**Figure 4:**
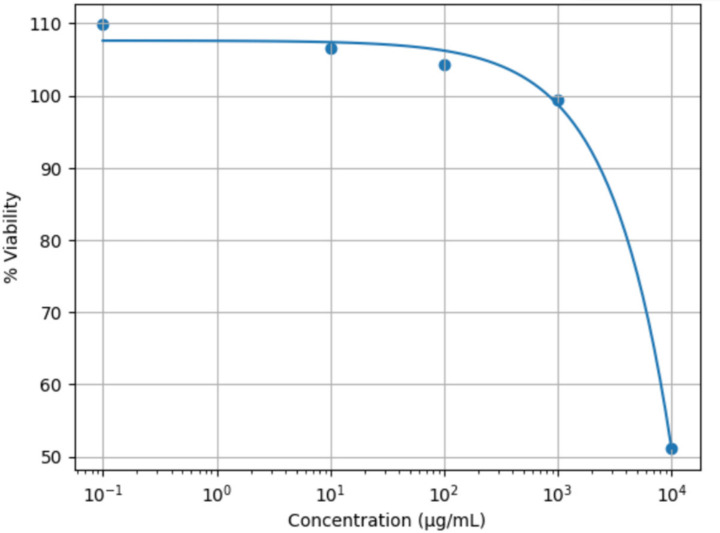
Cytotoxic effect against MDCK for different concentrations of HT.

The cytotoxic effect of HT on MDCK cells was evaluated using the MTT assay (Figure 4). At low and intermediate concentrations ranging from 0.1 to 10,000 µg/mL, HT did not exhibit any significant cytotoxicity. The cell viability remained close to or above 100%, with values of 106.49%, 104.27%, 99.43%, and 109.83%, respectively.

In contrast, a marked reduction in cell viability was observed at the highest concentration tested (10,000 µg/mL), where viability decreased to 51.09%, corresponding to a cytotoxicity of 48.91%.

The negative control exhibited 100% cell viability.

#### 3.2.2. Virucidal Effect of HT

**Figure 5:**
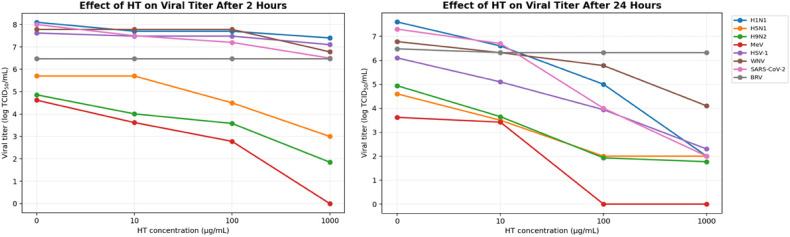
Effect of HT concentrations used on viral titers after adopted time of incubation: 2 h and 24 h.

The virucidal activity of synthetic HT was assessed against seven enveloped viruses—H1N1, H5N1, H9N2, MeV, HSV-1, WNV, and SARS-CoV-2—by quantifying the viral titers (log TCID_50_/mL) following exposure to increasing concentrations of HT (10–1000 µg/mL) for 2 and 24 h. After 2 h, most viruses exhibited modest reductions at lower concentrations, with more pronounced effects at 1000 µg/mL. MeV and H9N2 were particularly sensitive, with titers decreasing from 4.62 to 0 and from 4.86 to 1.85 log TCID_50_/mL, respectively. In contrast, HSV-1 and WNV showed minimal reductions at 10 and 100 µg/mL, with more substantial inhibition observed only at the highest dose. SARS-CoV-2 demonstrated a moderate response, with the titers decreasing from 8 to 6.5. Following 24 h of treatment, all viruses exhibited a marked concentration-dependent reduction in the viral load. For instance, the H1N1 and SARS-CoV-2 titers dropped from 7.6 and 7.3 to 2.0 log TCID_50_/mL, respectively, at 1000 µg/mL, while MeV became undetectable at higher concentrations (Figure 5).

**Figure 6:**
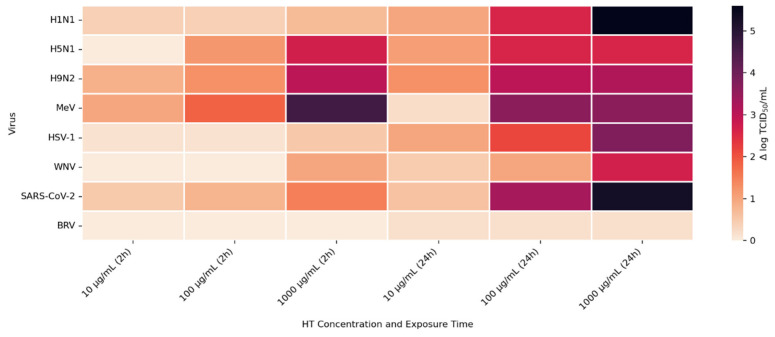
Heatmap representation of viral titer reduction (Δ log TCID_50_/mL) induced by hydroxytyrosol across different viruses, concentrations (10, 100 and 1000 µg/mL), and exposure times (2 h and 24 h).

To further quantify HT’s efficacy, viral titer reductions (Δ log TCID_50_/mL) were visualized using a heatmap (Figure 6). The results confirmed a dose- and time-dependent virucidal profile. At 10 µg/mL, most viruses showed mild inhibition after 2 h (Δ log ≤ 1), with slightly greater effects after 24 h, particularly for H9N2 (1.29), HSV-1 (1.0), and H5N1 (1.1). At 100 µg/mL, stronger reductions were observed, especially at 24 h, with SARS-CoV-2, MeV, and H9N2 showing Δ log values of 3.3, 3.32, and 3.01, respectively. The most pronounced virucidal effects occurred at 1000 µg/mL after 24 h, with Δ log reductions of 5.6 for H1N1, 5.3 for SARS-CoV-2, 3.8 for HSV-1, and over 3 for H9N2, MeV, and WNV. These findings indicate that H1N1, SARS-CoV-2, HSV-1, MeV and H9N2 were the most susceptible to HT, particularly at higher concentrations and longer exposure times, supporting its broad-spectrum, time-dependent virucidal potential.

Synthetic HT concentrations from 10 to 1000 µg/mL were applied to BRV under identical conditions as enveloped viruses for 2 h and 24 h to evaluate their susceptibility. The viral titers of bovine rotavirus proved completely resistant to synthetic HT because no decrease in viral quantity occurred at any of the tested concentrations or exposure durations. The BRV viral titers maintained a stable level of 6.48 log TCID_50_/mL throughout the entire 2-h exposure period at all the tested concentrations. The highest concentration of 1000 µg/mL failed to decrease the infectivity of the rotavirus. The viral titers of BRV showed no change after 24 h of treatment (Figure 5). The non-enveloped viruses showed no decrease in their ability to infect cells because their Δ log value remained at 0. The heatmap (Figure 6) shows the Δ log TCID50/mL evolution.

## 4. Discussion

Based on the previous literature and the known physicochemical properties of HT, we conducted in silico and in vitro investigations to evaluate HT’s antiviral activity, with a particular focus on its potential interference with lipid raft-mediated endocytosis in enveloped viruses.

Before conducting virtual screening, it is necessary to evaluate the compound based on Lipinski’s rule of five to ensure that it fulfills the key drug-likeness criteria [16,17]. This involves analyzing its molecular properties, with a particular focus on absorption, distribution, metabolism, and excretion (ADMET) [17], which are vital for assessing the potential as an effective drug candidate. In addition, the favorable physicochemical properties of HT, including a good membrane permeability and a high water solubility, were tested. Our ADME/toxicity results indicated a high gastrointestinal absorption and a low risk of metabolic interactions, making HT suitable for oral bioavailability. However, its limited permeability across biological barriers, particularly its Caco-2 and MDCK permeability, suggests challenges in systemic distribution. While HT exhibits a low systemic toxicity and a relatively low predicted acute toxicity (LD_50_ = 2820 mg/kg), moderate concerns about hepatotoxicity and ototoxicity need to be addressed in future development. Overall, HT shows promise as a virucidal compound, but further optimization is necessary to improve its bioavailability and minimize the toxicity risks.

Over the last decade, numerous in vitro studies have demonstrated the virucidal effect of HT on viruses like HIV-1, SIV, human and avian influenza viruses, and SARS-CoV-2. The present study confirms these findings with our strains and our synthetic HT. The mentioned enveloped viruses possess an envelope surrounding their capsid protein, suggesting that the mechanism of virucidal activity of HT might require the presence of the viral envelope, since HT is proven to be effective against enveloped viruses, but not against non-enveloped viruses such as bovine rotavirus and fowl adenovirus [5]. In line with these observations, our results showed no significant virucidal effect of HT against bovine rotavirus, which lacks a lipid envelope. Rotavirus particles are composed of a triple-layered protein capsid, without any host-derived lipid membranes or raft-associated components [13]. As HT may interact with lipid-associated structures and potentially affect the envelope integrity, the absence of such structures in the rotavirus may explains its resistance to HT treatments.

The envelope mainly consisted of a host-derived lipid bilayer membrane, in addition to a glycoprotein of virus origin [18]. It is known that the choice of which membrane the virus buds from and acquires its envelope from is often determined by intracellular trafficking and the specific accumulation of the viral envelope proteins at a particular time in the cell tropism [19]. Indeed, there are viruses that bud from the plasma membrane, like retroviruses and paramyxoviruses; those who bud from the endoplasmic reticulum, such as coronaviruses; and those who undergo transient envelopment and re-envelopment, like herpesviruses [19]. Importantly, although the viral envelope originates from a cellular membrane, its lipid composition is not identical to that of the donor membrane. Several enveloped viruses, including HIV-1, selectively enrich their envelope in phosphatidylserine (PS), sphingomyelin (SM), hexosylceramides and saturated phosphatidylcholine species, reflecting the concentration of lipid raft-associated components during budding [20]. This enrichment highlights the structural and functional importance of lipid rafts in the stability, entry and infectivity of enveloped viruses. This feature is particularly relevant when considering the antiviral activity of hydroxytyrosol. Since HT is known to disrupt lipid domains and interfere with raft-associated components, its inhibition is expected to primarily affect viruses whose infectivity depends on the integrity of these raft-enriched envelopes. Conversely, viruses that do not rely on such lipid microdomains, such as herpesviruses in the case of oleuropein (Ole), may display a reduced susceptibility [21]. At a mechanistic level, because the entry pathways, tropism and replication cycles differ among enveloped viruses, the precise mode of antiviral action of HT may vary between viral families [5].

The replication process of a rotavirus does not result in lipid membrane acquisition, which differentiates it from enveloped viruses. The virus structure depends only on its protein layers, which consist of VP2, VP6 and VP7 arranged in concentric fashion to create a stable non-enveloped virion [13]. The entry process of rotaviruses does not require lipid-raft-mediated fusion because it uses receptor-mediated endocytosis to enter cells. Upon contact with the cellular receptor, VP4 undergoes conformational changes such that the lipophilic domains of VP5*, usually concealed under VP8*, are exposed on the surface in a “post-penetration umbrella conformation”. Treating RV particles with trypsin appears to promote this transition and confer complete infectivity [13].

In contrast, no reductions in the viral titer were observed for bovine rotavirus following exposure to HT. As a non-enveloped virus, a rotavirus lacks a lipid membrane, which could potentially be viewed as a confounding factor. In the present study, HT was incubated with BRV prior to any trypsin treatment, allowing sufficient time for potential virucidal interactions to occur. Notably, a trypsin activation step (30 min at 37 °C) was performed only after the virus–HT incubation and immediately before titration and inoculation onto MA104 cells. Therefore, HT had the opportunity to exert its effect on the virus independently of trypsin. Despite this, no reduction in the rotavirus titer was observed following HT exposure, suggesting that the lack of activity is unlikely to result solely from trypsin interference. Instead, this finding may reflect an intrinsic limitation of HT against non-enveloped viruses. This interpretation is consistent with previous findings reported by Kentaro Yamada [5], who observed that HT exhibited antiviral activity against enveloped viruses, but not against non-enveloped viruses such as rotaviruses, despite the use of trypsin in the experimental protocol.

Taken together, these results support the hypothesis that the antiviral activity of HT is primarily associated with structural features of enveloped viruses. Nevertheless, the potential contribution of trypsin cannot be complexly excluded, and further investigations would be required to definitively rule out any interaction between trypsin and hydroxytyrosol.

Our molecular docking analyses revealed that hydroxytyrosol exhibits a strong in silico binding affinity for two key lipid raft components: ceramide and sphingomyelin. These lipids are well known for their roles in viral entry processes and the structural organization of viral envelopes. In the case of the influenza A virus, budding occurs at cholesterol-rich lipid rafts in the host plasma membrane, leading to an envelope composition highly enriched in sphingomyelin [22]. Biochemical studies have demonstrated that influenza virions contain higher levels of sphingolipids compared to the host cell membrane. An intact sphingomyelin biosynthesis pathway is essential for the proper trafficking of viral glycoproteins, such as hemagglutinin (HA) and neuraminidase (NA), to viral assembly sites, underscoring the critical role of sphingomyelin-rich microdomains in the assembly and budding of influenza virus particles [22]. Functional studies of influenza A have further shown that raft-associated lipids such as sphingomyelin and cholesterol are essential for the proper organization and incorporation of viral glycoproteins during virion formation [5]. Our in vitro screening results are consistent with these findings, as the highest Δ log TCID_50_/mL reduction was recorded against H1N1, reaching 5.6 at 1000 µg/mL of HT after 24 h of exposure. Similar enrichment in sphingomyelin has been observed in the viral envelopes of the measles virus (MeV) and West Nile virus (WNV), likely resulting from their assembly in lipid raft domains. In these viruses, sphingomyelin may contribute to membrane curvature and efficient budding [23].

Ceramide, on the other hand, is generated during the entry of several viruses through the hydrolysis of sphingomyelin by sphingomyelinases. For example, binding of MeV glycoproteins to the DC-SIGN/CD150 receptor complex activates sphingomyelinase, generating ceramide-enriched microdomains that cluster around entry receptors and facilitate membrane fusion [23]. Similarly, WNV infection leads to elevated intracellular ceramide levels, which appear to be involved primarily in signaling functions rather than virion structure. Importantly, the conversion of sphingomyelin to ceramide is necessary for WNV particle release, as inhibition of sphingomyelinase significantly reduces the production of infectious virions [24]. In the case of herpes simplex virus type 1 (HSV-1), ceramide’s role appears to be indirect, modulating the membrane composition by influencing the sphingomyelin content. Notably, the HSV-1 protein pUL21 has been shown to alter ceramide metabolism by activating CERT, a protein involved in inter-organelle lipid transport [25].

The docking results suggest that HT interacts with ceramide via key residues such as Tyr182 and Ile219, and with sphingomyelin via Phe163 and Ser167, suggesting that HT may disrupt viral entry by destabilizing the lipid raft integrity. By interfering with the structural and functional organization of these microdomains, HT could impair the early stages of viral infection, including attachment, fusion, and viral protein trafficking, ultimately reducing the viral efficiency.

SARS-CoV-2, like other coronaviruses, acquires its lipid envelope during budding from intracellular membranes, primarily the endoplasmic reticulum (ER), which are enriched in specific lipids such as phosphatidylcholine (PC) and phosphatidylinositol (PI). In contrast to viruses like HSV-1, the SARS-CoV-2 envelope contains lower levels of sphingomyelin (SM) and ceramide (Cer), a distinction that may influence the virus’s biophysical properties and sensitivity to antiviral compounds [26]. This lipid composition may partially explain the moderate virucidal activity observed for HT during short-term exposure, where a 2-h treatment with 1000 µg/mL results in a Δ log TCID_50_/mL reduction of 1.5. However, extending the exposure to 24 h significantly enhances HT’s efficacy, yielding a Δ log TCID_50_/mL reduction of 5.3 at the same concentration, suggesting that HT is active against SARS-CoV-2, but needs a higher dose and longer exposure. In terms of viral entry, SARS-CoV-2 utilizes its spike protein to bind to the angiotensin-converting enzyme 2 (ACE2) receptor, predominantly expressed on lung and intestinal epithelial cells, which are regions that are also rich in lipid rafts [27]. These microdomains play a critical role in organizing fusion platforms, and their role in coronavirus entry has been demonstrated in several models [28]. For instance, in Vero E6 cells, lipid rafts are essential for SARS-CoV entry, while in human coronavirus 229E, cholesterol depletion impairs viral entry by disrupting its interaction with the CD13 receptor [27,28]. Consistent with its non-enveloped nature, no measurable reduction in viral titers was observed for BRV under the same experimental conditions, reinforcing the notion that HT requires the presence of a lipid envelope to exert its virucidal effect.

Altogether, these findings underscore the importance of lipid raft integrity in SARS-CoV-2 infection and suggest that agents targeting membrane composition, such as HT, may hold therapeutic potential by interfering with virus–host membrane interactions.

The present results should be interpreted as indicative rather than statistically validated, as independent biological replicates were not performed. Although the consistent dose- and time-dependent trends and the differential response between enveloped and non-enveloped viruses support the coherence of the observed effects, future studies including biological replicates and formal statistical analyses will be required to confirm the virucidal efficacy quantitatively.

Although no reference virucidal positive control was included, the experimental design incorporated time-matched untreated virus controls at T0, 2 h and 24 h, enabling correction for potential spontaneous loss of viral infectivity during incubation. The maintenance of high viral titers in untreated controls supports the idea that the reductions observed in HT-treated samples were attributable to hydroxytyrosol rather than incubation-related decay. Moreover, because this study involved a heterogeneous multi-virus panel with distinct viral families and mechanisms, identification of a single universal positive virucidal control is not straightforward. Nevertheless, inclusion of an appropriate virucidal benchmark would further strengthen future studies.

The cytotoxicity was assessed on MDCK cells using the MTT assay and showed that hydroxytyrosol was well tolerated at the concentrations used in the antiviral experiments. Although quantitative assays were not performed on all the cell lines, no morphological signs of toxicity were observed in the treated cultures compared to the controls. These findings suggest that the antiviral effects are unlikely to result from nonspecific cytotoxicity, although further evaluation across additional cell lines would be beneficial.

Although the present study demonstrates a consistent dose- and time-dependent virucidal effect of hydroxytyrosol, the use of only three discrete concentrations limits the ability to derive quantitative pharmacological parameters such as the IC_50_ or EC_50_ values. Future studies that include a wider concentration range and additional replicates will be necessary to establish formal dose–response relationships and allow for accurate potency comparisons.

The proposed mechanism involving viral envelope disruption is supported by indirect evidence, including selective activity against enveloped viruses, a lack of effect on rotaviruses, and predicted interactions with lipid raft components. However, direct structural confirmation (e.g., electron microscopy or membrane assays) was not performed. In addition, alternative mechanisms, such as differences in receptor usage or entry pathways, cannot be excluded. Therefore, this mechanism should be considered a plausible hypothesis requiring further validation.

## 5. Conclusions

This study integrated both in silico and in vitro approaches to explore the virucidal activity of HT against enveloped and non-enveloped viruses, and it provides preliminary evidence supporting HT’s potential to reduce viral infectivity. Our findings suggest that the observed activity may be associated with interactions between HT and lipid raft components, such as ceramide and sphingomyelin, based on molecular docking results and previously reported viral lipid compositions, with higher levels potentially enhancing the HT-mediated alternation of the viral particle integrity. The absence of virucidal activity observed against bovine rotavirus further supports the hypothesis that viral envelope-associated structural features may influence the susceptibility to HT.

## Figures and Tables

**Table 1:** Binding energy and site of interaction of hydroxytyrosol with the different targets studied.

Target	Binding Energy (Kcal/mol)	Site Interaction for HT–Target
Ceramide	−6	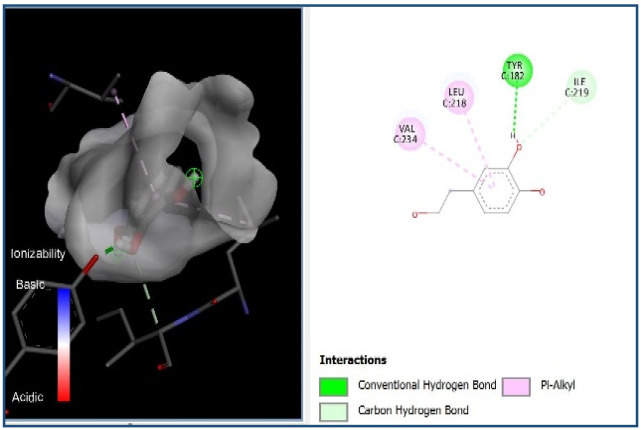
Sphingomyelin	−5.9	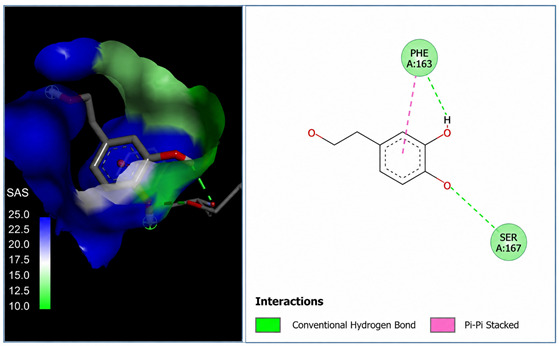
Phosphatidylinositol	−5.8	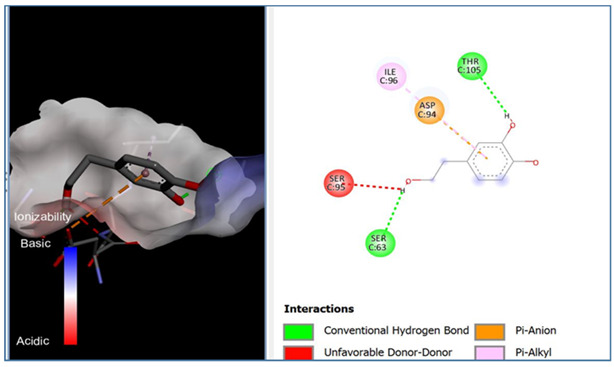
Cholesterol	−5.6	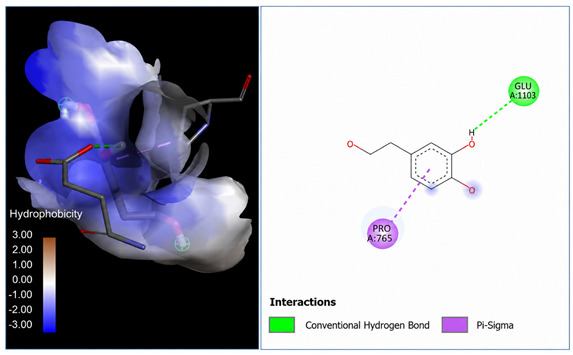
Phosphatidylcholine	−5	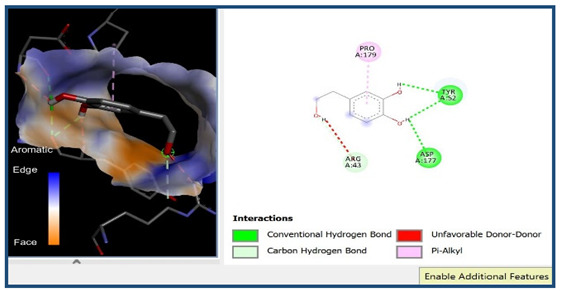
Lysophosphatidic Acid	−4.5	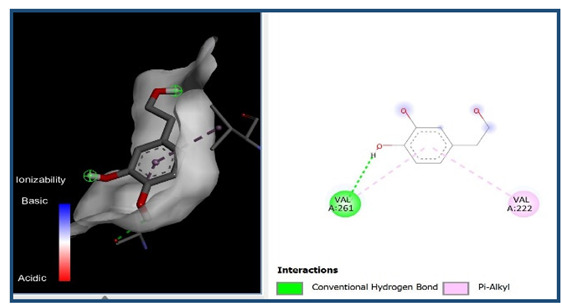
Lysophosphatidylethanolamine	−3.2	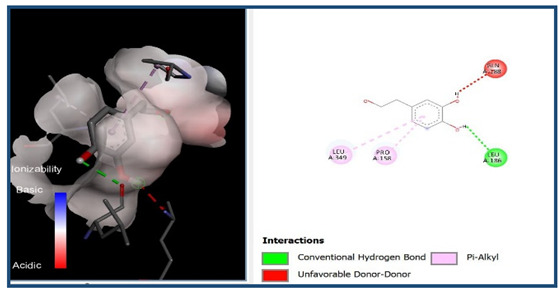

**Table 2:** Assessment of ADME kinetics of HT by using SwissADME software.

Compound	Physicochemical Properties	Lipophilicity	Water Solubility	Pharmacokinetics	Drug-Likeness
**HT**	Formula: C_8_H_10_O_3_ MW: 154.16 g/mol Nrb^⁎^: 2 NHA^⁎^: 3 NHD^⁎^: 3 TPSA: 60.69 Å^2^	Log-Po/w (iLOGP): 1.28	Solubility: 3.75 × 10^1^ mg/mL Class: Very soluble	GI absorption: High BBB: No P-gp substrate: No CYP1A2 inhibitor: No CYP2C19 inhibitor: No CYP2C9 inhibitor: No CYP2D6 inhibitor: No CYP3A4 inhibitor: No	Lipinski: Yes; 0 violation Ghose: No; 1 violation Veber: Yes Egan: Yes Muegge: No; 1 violation

⁎ **Nrb** refers to: *Number of rotatable bonds*; **NHA** for *Number of hydrogen bond acceptors* and **NHD** refers to *Number of hydrogen bond donors*.

**Table 3:** ADMET analysis of HT *via* ADMET Lab 2.0.

Characteristics	Value	Prediction *	Toxicity Class
Caco-2 Permeability	−4.997	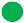	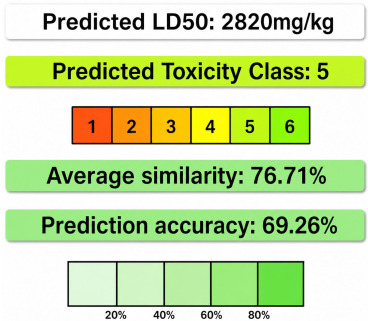
MDCK Permeability	0	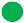	
HIA	-	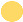	
Plasma Protein Binding	26.6%	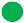	
Blood–Brain Barrier Penetration	---	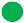	
HLM Stability	+	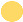	
Skin Sensitization	0.992	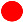	
Carcinogenicity	0.444	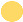	
Eye Corrosion	0.958	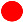	
Eye Irritation	0.998	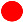	
Respiratory	0.216	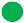	
Human Hepatotoxicity	0.377	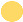	
Drug-Induced Nephrotoxicity	0.057	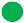	
Drug-Induced Neurotoxicity	0.041	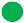	
Ototoxicity	0.553	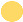	
Hematotoxicity	0.097	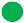	
Genotoxicity	0.076	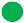	
NR-AhR	--	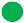	
NR-AR	---	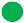	
NR-AR-LBD	---	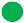	
NR-Aromatase	---	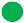	
NR-ER	---	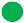	
NR-ER-LBD	-	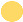	
NR-PPAR-gamma	---	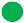	
SR-ARE	+	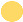	
SR-ATAD5	---	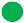	
SR-HSE	--	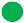	
SR-MMP	--	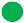	
SR-p53	---	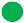	

* For the classification endpoints, the prediction probability values were transformed into six symbols: 0–0.1 (---), 0.1–0.3 (--), 0.3–0.5 (-), 0.5–0.7 (+). Additionally, the corresponding relationships of the three labels are as follows: Green «excellent»; Yellow «medium»; Red «poor».

## Data Availability

The original contributions presented in this study are included in the article/Supplementary Material. Further inquiries can be directed to the corresponding author.

## Supplementary Materials

The following supporting information can be downloaded at: https://www.mdpi.com/article/10.3390/cimb48050481/s1.
