# Supplementary material for: Exploring Hydroxytyrosol as a Promising Virucidal Agent: In Silico and In Vitro Insights into Enveloped Viruses
Source: Curr Issues Mol Biol. 2026 May 5;48(5):481. doi: 10.3390/cimb48050481 (PMC13206310; doi:10.3390/cimb48050481)
Supplement: Supplementary file 1 [file cimb-48-00481-s001.zip › cimb-4273494-supplementary.pdf]

Table S1: Bioinformatics Tools used in this *in-silico* screening

***In-silico* Tool**	**Type**	**Prediction**	**Availability**	**Open source License**
**PyRx**	Software	Virtual Screening (docking and scoring)	Freely available	http://pyrx.sourceforge.net/
**Open Babel version 2.3**	Software	Chemical Tool Box to search, convert, analyze, or store data from molecular modeling, chemistry, and solid-state materials.	Freely available	http://openbabel.org.
**BIOVIA Discovery Studio Visualizer (version 2020)**	Software	Target preparation<br>Visualization of Ligand-protein interaction.	Freely available	https://discover.3ds.com/discovery-studio-visualizer-download
**RCSB PDB**	Database	Efficient Tool to explore, visualize, and analyze the experimentally determined 3D structure of macromolecules (mainly proteins and nucleic acid) by X-ray crystallography or NMR spectroscopy.	Freely available	http://pdb.org/
**PuChem NCBI**	Open chemistry Database	Collect data about molecules such as chemical structure, Toxicity, and Physicochemical properties via CID (Compound ID)	Freely available	https://pubchem.ncbi.nlm.nih.gov/
**DrugBank**	Open chemistry Database	vital resource for your pharmaceutical research, offering comprehensive and reliable drug data	free access	https://go.drugbank.com/
**ProTox II**	Webserver	Prediction of Toxicity and ADME of Chemicals	Freely available	http://tox.charite.de/tox
**SwissADMET**	Webserver	Estimation in-silico of physico-chemistry, pharmacokinetics, drug-likeness, and medicinal chemistry friendliness of small compounds.	free access	http://www.swissadme.ch/
**PreADMET Lab 2.0**	Webserver	systematical evaluation of ADMET properties	free	https://admetmesh.scbdd.com/
**LipidMaps**	Webserver	A free, open-access lipidomics resource	free	https://lipidmaps.org/
**SphinGOMAP©**	Webserver	web-based biosynthetic pathway map of sphingolipids and glycosphingolipids	free	https://lipidmaps.org/resources/sphingomap

Table S2: Summary of the molecular and chemical characteristics of the HT.

IUPAC Name	Molecular Summary	Chemical Taxonomy	2D Structure	3D Structure
**4-(2-hydroxyethyl)benzene-1,2-diol**	$C_8H_{10}O_3$<br>154.16 g/mol<br>PubChem ID : 82755<br>DrugBank Accession Number: DB12771 SMILES OCCC1=CC= (O)C(O)=C1	This compound belongs to the class of organic compounds, Super Class Benzenoids, Class Phenols. These compounds are known as tyrosols. These are organic compounds containing a phenethyl alcohol moiety that carries a hydroxyl group at the 4-position of the benzene group.	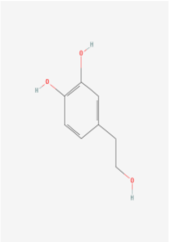	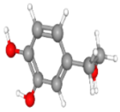

Table S3: The Code PDB of the selected targets

Target	ID PDB	Systematic name	LM_ID
**ceramide**	9eot	CerP(d18:1/2:0)	LMSP02050014
**sphingomyelin**	8ijq	N-acyl-sphing-4-enine-1-phosphocholine	LMSP03010000
**phosphatidylinositol**	5eut	1,2-dihexadecanoyl-sn-glycero-3-phospho-(1'-myo-inositol)	LMGP06010007
**Cholesterol**	7df8	cholest-5-en-3β-ol	LMST01010001
**phosphatidylcholine**	1ln1	1,2-diacyl-sn-glycero-3-phosphocholine	LMGP01010000
**Lysophosphatidic acid**	4z34	1-acyl-sn-glycero-3-phosphate	LMGP10050000
**Lysophosphatidylethanolamine**	2dde	-	-
